# Elastic instabilities in planar elongational flow of monodisperse polymer solutions

**DOI:** 10.1038/srep33029

**Published:** 2016-09-12

**Authors:** Simon J. Haward, Gareth H. McKinley, Amy Q. Shen

**Affiliations:** 1 Okinawa Institute of Science and Technology, Onna-son, Okinawa, 904-0495, Japan; 2 Massachusetts Institute of Technology, 77 Massachusetts Avenue, Cambridge, MA 02139, United States

## Abstract

We investigate purely elastic flow instabilities in the almost ideal planar stagnation point elongational flow field generated by a microfluidic optimized-shape cross-slot extensional rheometer (OSCER). We use time-resolved flow velocimetry and full-field birefringence microscopy to study the behavior of a series of well-characterized viscoelastic polymer solutions under conditions of low fluid inertia and over a wide range of imposed deformation rates. At low deformation rates the flow is steady and symmetric and appears Newtonian-like, while at high deformation rates we observe the onset of a flow asymmetry resembling the purely elastic instabilities reported in standard-shaped cross-slot devices. However, for intermediate rates, we observe a new type of elastic instability characterized by a lateral displacement and time-dependent motion of the stagnation point. At the onset of this new instability, we evaluate a well-known dimensionless criterion *M* that predicts the onset of elastic instabilities based on geometric and rheological scaling parameters. The criterion yields maximum values of *M* which compare well with critical values of *M* for the onset of elastic instabilities in viscometric torsional flows. We conclude that the same mechanism of tension acting along curved streamlines governs the onset of elastic instabilities in both extensional (irrotational) and torsional (rotational) viscoelastic flows.

Extensional kinematics occur locally in all flows that possess streamwise velocity gradients, including flows through intersections (like T- or Y-shaped junctions), flows through contractions or expansions, and flows around obstacles such as sedimenting particles. Many industrial and technological processes involve the flow of viscoelastic polymeric fluids under conditions of strong extensional deformation, resulting in a complex rheological response from the fluid. The maximum rates at which many processing operations involving polymeric fluids can be carried out are restricted by the onset of elastic instabilities, which would be entirely unexpected for the equivalent flow of a Newtonian fluid^1^,^2^,^3^. Understanding of the conditions resulting in the onset of elastic instabilities in viscometric shearing flows is now quite advanced^4^,^5^,^6^, however the same is not true for extension-dominated or “shear-free” flows, which are much more difficult to study under well-controlled and well-defined conditions^7^,^8^. Gaining a complete understanding of the factors causing the onset of elastic instabilities in arbitrary flow kinematics will be of benefit to the optimization of widespread applications and processes including extrusion, fiber-spinning, blow-moulding, inkjet printing, lab-on-chip design and laboratory microfluidics experiments^3^.

The cross-slot device is a common flow geometry that is widely utilized for generating a controllable planar extensional flow field. It consists of mutually bisecting rectangular channels with two opposing inlets and two opposing outlets and, under ideal conditions, the symmetry of the geometry results in the occurrence of an isolated stagnation point at the precise center of the flow field^9^. Planar elongation occurs as fluid elements accelerate away from the stagnation point along the axis of the outlet channels. This extensional flow field has proven itself extremely useful in laboratory applications^9^. In particular, cross-slot devices have yielded significant insights into the stretching dynamics of polymers in dilute solution under strong elongational flow fields^10^,^11^,^12^,^13^. Experiments with solutions of flexible polymers have confirmed that as the strength of the extensional flow is increased such that the magnitude of the Weissenberg number exceeds a critical value given by 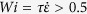 (where *τ* is the characteristic relaxation time of the fluid and 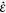 is the applied elongation rate), polymer molecules in the region of the stagnation point can undergo a conformational change from a random coil to a highly stretched state, known as the coil→stretch transition^14^,^15^,^16^,^17^. This has been shown by measuring the resulting optical anisotropy, or flow-induced birefringence, in the polymer solution using polarized light techniques^10^,^18^,^19^,^20^, and also by direct observations of single molecules of fluorescently-labelled DNA unraveling at the stagnation point^11^,^12^. The stretching of polymer molecules at the stagnation point and the entropic elasticity driving their relaxation as they are advected downstream results in the formation of an elastic strand localized along the outflowing symmetry plane of the cross-slot, a so-called “birefringent strand”^10^,^21^,^22^,^23^. Within these regions, the highly extended polymers strongly resist additional deformation, elastic tensile stresses dominate and lead to a non-Newtonian increase in the local extensional viscosity of the fluid. The effective viscosity within the elastic strand of highly-aligned polymer can be orders of magnitude greater than the viscosity of the fluid surrounding the strand, in which the polymers are only weakly deformed from their equilibrium coil configurations^21^,^20^. The extensional viscosity of the elastic strand can be deduced by measurements of the non-linear increase in the bulk pressure drop across the cross-slot device as 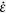 is increased, or alternatively by measuring the local birefringence and invoking the stress-optical rule (SOR)^19^,^20^,^24^,^25^,^26^,^27^. The local increase in extensional viscosity within the birefringent strands is so great that they can even be modeled to a good approximation as internal elastic boundary layers in the flow field^21^. This can cause severe perturbations to the flow field compared with the Newtonian case^20^,^21^,^28^,^29^,^30^,^31^. Feedback between the polymer elongation in the strand and the resulting flow field modification can give rise to a variety of theoretically predicted and experimentally observed elasticity-influenced flow instabilities^22^,^30^,^32^,^33^,^34^,^35^,^36^,^37^. Of particular relevance to the present study is a flow asymmetry, first reported by Gardner *et al*.^30^ that can occur for viscoelastic flows in cross-slot devices at high deformation rates.

Over the past decade, there has been an increasing interest among the experimental and computational fluid dynamics and rheology communities in the flow asymmetry observed by Gardner *et al*.^30^ as an example of a “purely-elastic” flow instability^34^,^35^,^38^,^39^,^40^,^41^,^42^,^43^. This symmetry-breaking flow bifurcation occurs when inertia is negligible (i.e. the Reynolds number, *Re*, is low) but elastic effects, as characterized by the Weissenberg number (*Wi*), become significant. The instability is characterized by the unequal division of the inlet flow between the two outlet channels of the cross-slot. Although first reported in the 1980’s^30^, study of this phenomenon has only proceeded in earnest since the advent of widely accessible techniques for the fabrication of microfluidic devices^44^. Since 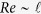 and 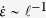, the inherently small length scales 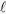 of microfluidic devices allow fluids to be deformed at high rates while inertia remains low^45^, and give ready access to regimes of very high elasticity *El* = *Wi*/*Re* at which elastic instabilities become prevalent^3^,^46^,^47^,^48^,^49^,^50^.

Purely elastic instabilities, i.e. those arising when inertial forces are negligible compared with elasticity, have been reported for viscoelastic fluids in a wide variety of flow configurations^2^,^3^,^4^,^6^. An example pertinent to the present investigation is the flow of polymer solutions into abrupt contractions, which is a widely studied problem due to its great industrial relevance in polymer processing^51^,^52^,^53^,^54^,^55^,^56^. In this case a rich sequence of instabilities can be observed as the flow rate through the contraction is increased. These instabilities have been characterized extensively over a wide range of *Wi* and *Re* by varying fluid properties and channel dimensions in microfluidic planar abrupt contraction geometries^46^,^47^,^57^,^58^,^59^. For fluids of high elasticity (*El* > 1), the Weissenberg number is the dominant parameter controlling the initial onset of instability. For low *Wi*, the flow is steady and appears Newtonian-like, but as the Weissenberg number is increased streamlines may begin to diverge as they approach the contraction throat, a feature which is often soon followed by the formation of “lip-vortices” at the reentrant corners and the onset of unsteady flow^46^,^47^,^57^,^58^,^59^. Further increases in *Wi* are usually associated with the formation of vortices in the salient corners upstream of the contraction throat, which may grow large distances upstream as the Weissenberg number is progressively increased. Here, depending on the elasticity number *El*, various scenarios are possible: the upstream corner vortices may remain steady and symmetric, or they may grow asymmetrically and may oscillate in size either periodically or aperiodically^46^,^47^,^57^,^58^,^59^.

Despite being widely studied and well-characterized, gaining a deeper understanding of the underlying physical mechanism of the onset of elastic instabilities in the abrupt contraction geometry has been elusive. The main reasons for this are the complex mixed kinematics of the flow field (which contains both strong shear at the walls and strong non-homogeneous elongational components as fluid accelerates into the contraction), combined with the large number of variable geometric parameters that can affect the instability. Far more success at understanding the onset conditions of elastic instabilities in polymer solutions has been achieved by examining well-defined, viscometric shearing flows, such as those generated by the Taylor-Couette^60^,^61^, the cone-plate^62^,^63^,^64^ and the plate-plate^62^,^63^,^65^ geometries (see extensive reviews provided by Larson^2^, Shaqfeh^4^ and Muller^6^). Such geometries are of great importance as they are the most frequently used devices for characterizing the rheology of complex fluids on rotational rheometers. Therefore understanding the critical conditions that result in flow instability is vital since this bounds the upper limit of the measurement range of the rheometer. The culmination of these studies of viscometric torsional flows via experiment, theory, simulation and linear stability analysis has been the development of a universal criterion for the onset of elastic instabilites, which couples streamwise normal stresses with the curvature of streamlines:


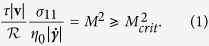


Here 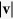 is the magnitude of the local flow velocity, 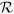 is the local radius of curvature of a streamline, *σ*_11_ is the normal stress in the streamwise direction, *η*_0_ is the zero shear rate viscosity of the fluid, *τ* is the characteristic relaxation time of the fluid, and 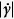 is the magnitude of the local deformation rate^5^,^66^. The first term on the left can be thought of as a ratio of lengthscales: the product 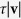 describes a distance over which perturbations to the flow field due to elastic stresses relax – if this distance is large relative to the streamline radius of curvature, the flow becomes prone to instability. However, the magnitude of the elastic stress acting along the curved streamline is also important, and this is accounted for by the coupling with the second term on the left. For a fixed geometry, increases in fluid flow rate generally lead to proportionate increases in both the local velocity and the shear rate. In addition, since for a given fluid *η*_0_ and *τ* are material constants, it is apparent from Eq. 1 that the most important parameters governing the magnitude of *M* are 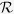 and *σ*_11_. It is important to note that Eq. 1 does not anticipate a numerical value for *M*_*crit*_ but only describes how it should scale with rheological and geometric parameters. The scaling has been shown to hold in the Taylor-Couette and the cone-plate geometry providing values of 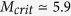 and 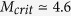, respectively^5^. McKinley *et al*.^5^ have also demonstrated expected scalings for some more complex two-dimensional flows, arriving at values of 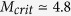 for the lid-driven cavity, and 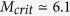 for flow past a confined cylinder. McKinley *et al*.^5^ also consider the planar contraction geometry and point out that the curvature of streamlines depends on the contraction ratio 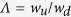, where *w*_*u*_ and *w*_*d*_ are the upstream and downstream channel widths, respectively. Therefore, for a given viscoelastic fluid, contraction geometries with higher values of 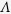 should be more prone to instability, which appears to be consistent with experimental results obtained in microchannels^59^. This highlights an interesting point that is in fact clear by inspection of Eq. 1: if the contraction ratio 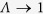, then the geometry becomes a straight planar channel, 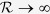 and *M* becomes identically equal to zero. Whether it is possible to observe elastic instabilities for viscoelastic fluids flowing in an infinitely long straight channel without some external perturbation being imposed is extremely challenging to test experimentally^67^ and is a matter of current debate, with some theoretical works indicating that nonlinear instability is still possible in the absence of any streamline curvature^68^.

Returning to the case in point of the cross-slot flow asymmetry, recently Cruz *et al*.^43^ have attempted to spatially evaluate the instability criterion *M* as a function of the applied *Wi* in cross-slot devices by means of numerical simulations performed with the upper-convected Maxwell (UCM) and simplified Phan-Thien Tanner (sPTT) viscoelastic constitutive models. These simulations were performed in geometries with sharp square reentrant corners at the channel intersections (which we will refer to from now on as “standard-shaped” cross-slot devices). Near the corners of such devices, finite elastic stresses are generated, which are of lower magnitude than at the stagnation point, however the streamline curvature near the corners is large and the flow velocity there is much higher than it is close to the stagnation point. Cruz *et al*. found that the highest values of *M* occur near the corners of the flow geometry and suggest that these are the primary instability-driving regions in the flow field, as opposed to the central stagnation point^43^. This supports the earlier work of Rocha *et al*. 39 who found the onset of the flow asymmetry was delayed to higher *Wi* if the corners of the standard-shaped cross-slot were rounded off.

However, ideal planar elongational flow, as described by the streamfunction 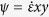, has hyperbolic streamlines whose curvature decreases continuously with increasing distance from the stagnation point and does not possess the sharp re-entrant corners of the standard-shaped cross-slot device. In this case the highest curvature is along streamlines passing close to the stagnation point, which must turn sharply through 90°. Numerous experiments invloving flow-induced birefringence measurements in stagnation point devices^20^,^27^,^32^,^37^,^69^, as well as simulations^35^,^36^,^39^ and theory^22^,^33^,^70^,^71^, show clearly that the birefringent strand of fluid carries high elastic stresses in a band of finite width about the stagnation point. Thus, close to the stagnation point, conditions exist for non-zero values of the *M* criterion to be possible, and this was shown by Öztekin *et al*.^72^ using simulations of the Oldroyd-B model in planar stagnation point flow. In terms of experiment, the microfluidic optimized-shape cross-slot extensional rheometer (OSCER) device (see Fig. 1) has been shown to generate an excellent approximation to the ideal streamfunction describing planar elongation^9^,^27^,^73^. Flows of highly elastic polymer solutions in the OSCER device indeed exhibit flow asymmetries of very similar appearance to those observed in the standard-shaped cross-slot device^37^. However, until now no experimental evaluation of the elastic instability criterion *M* has been performed in either the standard or the optimized-shape cross-slot type devices.

In this work we perform a detailed experimental study of the onset of elastic flow instabilities in the well-defined hyperbolic flow field within the OSCER geometry. We use a series of well-characterized nearly monodisperse polymer samples dissolved in a thermodynamically ideal *θ*-solvent, which we anticipate will be amenable to comparison with future numerical simulations. We use micro-particle image velocimetry (*μ*-PIV) to characterize the flow field and quantitative flow-induced birefringence measurements to quantify the stress fields in the polymer solutions as the imposed flow rate is progressively increased until the flow becomes unstable. Our detailed, time-resolved *μ*-PIV measurements reveal that the flow asymmetry in the OSCER geometry does not occur spontaneously but rather represents the later stage of development of an instability that begins at a much lower *Wi* (in a manner similar to the instability progression observed in the planar contraction geometry, described above). We evaluate the magnitude of the elastic instability criterion *M* at the onset of the first signs of instability (which corresponds to a lateral displacement and quasiperiodic lateral motion of the stagnation point) and we obtain maximum values of *M* in localized regions close to the stagnation point, as shown numerically by Öztekin *et al*.^72^ In the almost ideal planar elongational flow field provided by the OSCER device, we find good agreement with the scaling suggested by Eq. (1) and we obtain an estimate of *M*_*crit*_ for the onset of the first elastic instability that is comparable with values obtained previously in viscometric torsional shearing flows.

## Results

**Figure 1:**
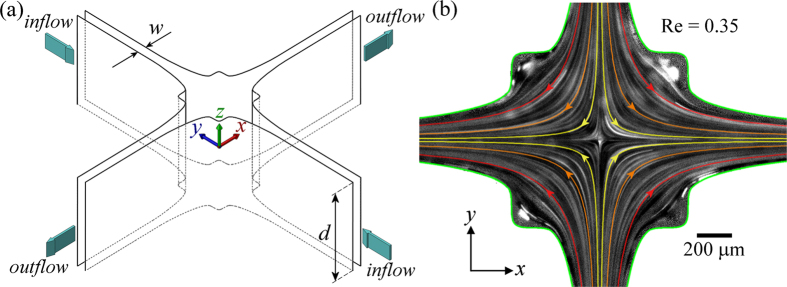
(**a**) 3D drawing of a portion of the Optimized Shape Cross-slot Extensional Rheometer (OSCER) device, indicating the principal channel dimensions (width *w* = 200 *μ*m, depth *d* = 2 mm) and the coordinate system with origin at the geometric center. (**b**) Streak photograph obtained from fluorescent tracer particles in a Newtonian fluid at *Re* = 0.35. Superimposed colored hyperbolae represent streamlines expected for ideal planar elongational flow and the arrows indicate the flow direction (inflow along ±*y*, outflow along ±*x*).

Flow experiments are performed in the microfluidic OSCER device^9^,^27^,^37^,^73^,^74^,^75^ illustrated schematically in Fig. 1a. The device has a shape that has been determined by a numerical optimization procedure^27^,^73^,^75^ in order to provide a close approximation to ideal planar elongational flow over a wide region of the flow field surrounding the central stagnation point. The fidelity of the flow field has been confirmed experimentally^9^,^27^,^75^ and is illustrated qualitatively in Fig. 1b, which shows a streak image obtained for low Reynolds number flow of a Newtonian fluid compared with theoretical hyperbolic streamlines obtained using the ideal streamfunction, 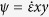. Test fluids are pumped through the OSCER device at controlled volume flow rates, *Q*, using four high precision syringe pumps (neMESYS, Cetoni GmbH). Two pumps simultaneously inject fluid at equal rates into the two opposing inlets while an additional two pumps withdraw fluid simultaneously at an equal and opposite rate from the two diametrically-opposed outlets. Syringe volumes are selected to ensure minimal pulsation in the resulting flow; the pumps displace fluid at a rate of at least 600 increments per second even for the lowest applied *Q*

### Newtonian flow characterization in the OSCER

Control experiments involve pumping the Newtonian solvent dioctyl phthalate (DOP) through the OSCER device over a range of applied flow rates and using micro-particle image velocimetry (*μ*-PIV)^76^,^77^,^78^ to confirm the expected characteristics of the flow field at Reynolds numbers spanning the range covered in later experiments with viscoelastic polymer solutions. The Reynolds number here is defined by 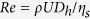, where 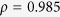 g mL^−1^ and *η*_*s*_ = 59 mPa s are the density and viscosity of the DOP, respectively, 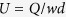 is the average flow velocity, and 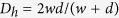 is the hydraulic diameter; *w* = 200*μ*m and *d* = 2 mm are the characteristic width and depth of the channel, respectively (see Fig. 1a).

**Figure 2:**
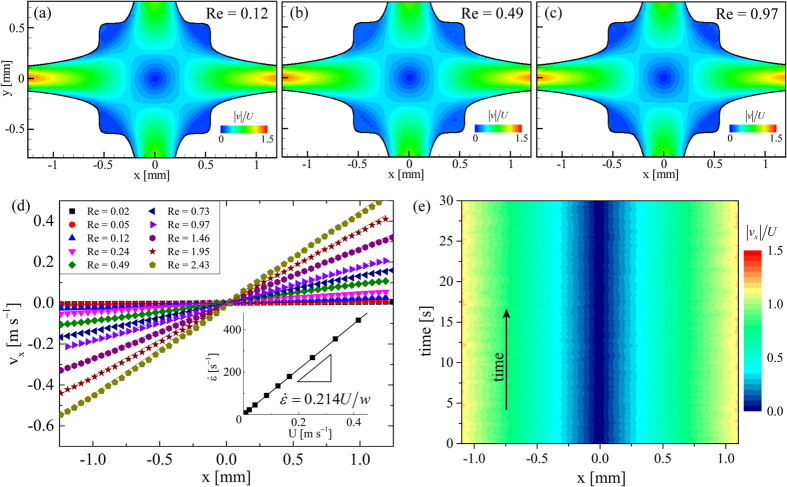
Control experiments to characterize the flow field in the OSCER geometry using the pure Newtonian solvent (DOP): **(a–c)** Normalized velocity magnitude fields obtained over a range of imposed *Re* show good self-similarity. (**d**) *x*-component of the velocity *v*_*x*_(*x*) measured along *y* = 0 shows proportionality at each imposed *Re*, i.e. a uniform velocity gradient. The correspondingly-colored lines passing through each data set are linear fits to the data through the origin, from which the velocity gradient at each imposed flow rate is obtained. Inset shows the streamwise velocity gradient along *y* = 0 (i.e. 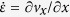) as a function of the average flow velocity *U*, displaying the expected linearity. (**e**) Space-time diagram showing the magnitude of *v*_*x*_(*x*, *t*) along *y* = 0 normalized by *U* at *Re* = 0.49, demonstrating the steadiness of the flow field over a 30 s time period (data captured at 4 Hz).

Over a wide range of *Re*, the Newtonian flow field shows good self-similarity, as exemplified by the normalized velocity magnitude fields shown in Fig. 2a–c. These are ensemble-averaged over 20 individual velocity fields captured over a 5 s period and show a centrally-located stagnation point and a velocity magnitude that continuously increases with distance from the stagnation point along the flow axes. Profiles of the *x*-component of the velocity along the *x*-axis (i.e. 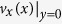) are extracted from such velocity fields and are shown in Fig. 2d. At each applied flow rate (or *Re*), *v*_*x*_ is proportional to *x*, i.e. the velocity gradient 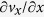 along *y* = 0 is constant over the measured range of *x*. This velocity gradient 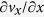 defines the elongation rate 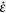 imposed on fluid elements passing through the OSCER device. The inset to Fig. 2d shows the relationship between the measured value of 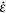 and the average imposed flow velocity *U*, which provides the following best linear fit :


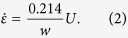


The constant of proportionality in Eq. 2 (0.214/*w*) is close to the expectation from two-dimensional (2D) numerical simulations (0.2/*w*) and the discrepancy is consistent with the finite aspect ratio of the experimental OSCER device (*α* = *d*/*w* = 10)^27^. Time-resolved *μ*-PIV measurements with the solvent confirm the temporal stability of the flow field in the Newtonian case. Velocity fields are collected at a rate of 4 Hz over a 30 s time period and profiles of 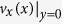 are extracted from each field. Figure 2e shows a space-time diagram composed of such velocity profiles for the case of Newtonian flow at *Re* = 0.49, and shows clearly how the stagnation point remains centrally-located and that spatio-temporal velocity fluctuations are low (at any position along the *y*-axis, rms velocity deviations over time are 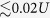).

**Figure 3:**
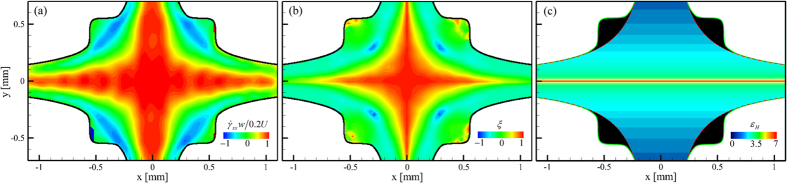
Spatially-resolved characterization of the Newtonian flow field in the OSCER device. (**a**) Strain rate field for the flow of DOP at *Re* = 0.49. (**b**) Flow type parameter for the flow of DOP at *Re* = 0.49. (**c**) Fluid Hencky strain computed assuming ideal hyperbolic streamlines within the hyperbolic region marked by dashed red lines.

The local components of the velocity fields (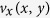 and 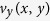) obtained from the *μ*-PIV experiments can be used to locally evaluate the components of the deformation rate 
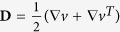
 and vorticity 
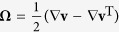
 tensors. In Fig. 3a we show the 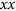 component of the deformation rate tensor (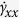) in normalized form. This was evaluated using the velocity field shown in Fig. 2b for the flow of the DOP solvent at *Re*  =  0.49. The result agrees very well with a 2D numerical prediction obtained for Newtonian creeping flow and illustrates the homogeneity of the flow field over the central region of the geometry^27^. It is also possible to locally evaluate the flowtype parameter, 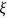 according to the criterion of Astarita^79^,^80^,^81^. The flowtype parameter is defined as:


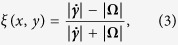


where 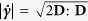 and 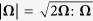 are the magnitudes of the deformation rate and vorticity tensors, respectively.

The flowtype parameter varies between −1 and 1, with values of −1 corresponding to purely rotational kinematics, values of 0 corresponding to purely shearing kinematics and values of +1 corresponding to purely extensional kinematics. Figure 3b shows the local flowtype parameter computed from the velocity field shown in Fig. 2b. It is clear that the flowtype is dominated by purely extensional kinematics along the flow axes and over a wide symmetrical region surrounding the central stagnation point.

It is also of value to consider the strain applied to fluid elements as they flow through the OSCER geometry. An estimate of the strain can be readily computed by assuming that fluid elements follow hyperbolic streamlines within the numerically optimized region of the flow geometry, i.e. over the domain spanning 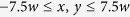 about the stagnation point^27^. Fluid elements enter this domain at initial locations given by 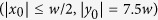 and exit the domain at final locations given by 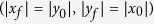. If a fluid element enters this domain at a position given by (*x*_0_,*y*_0_), the Hencky strain that the fluid element has accumulated at any subsequent position (*x*,*y*) along the streamline is given by 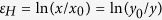. The result of this calculation performed over the entire domain is shown in Fig. 3c (cropped to the experimental field of view). The Hencky strain is constant along *x* and varies along *y* as 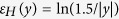 (with *y* in mm). The strain is sharply peaked about the *y* = 0 axis, where theoretically the strain becomes infinite. For |*y*| ≤ 1.5 *μ*m, *ε*_*H*_ exceeds 6.9 units. For |*y*| ≤ 1 *μ*m (which corresponds to the spatial resolution of our imaging system), *ε*_*H*_ ≈ 7.3 units.

### Viscoelastic test fluid characterization

Solutions of low-polydispersity atactic-polystyrene (a-PS) in the room-temperature (22 °C) *θ*-solvent DOP^82^ are prepared at molecular weights *M*_*p*_ = 6.9 and 16.2 MDa (denoted hereonwards as PS7 and PS16, respectively) and over a range of concentration 0.035 ≤ *c *≤ 0.14 wt.%. This polymer-solvent system is extremely well characterized and details of the molecular parameters of the two a-PS samples in the DOP solvent are provided in Table 1.

**Figure 4:**
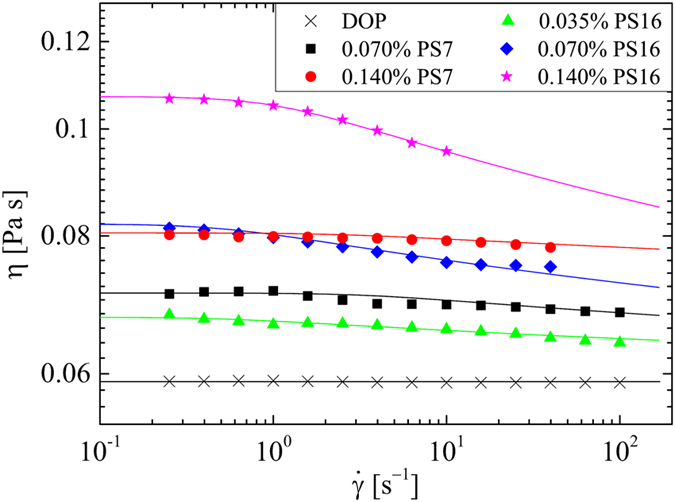
Steady flow curves of viscosity *η* as a function of the imposed shear rate 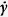 for the Newtonian solvent (DOP) and for the various polystyrene-based test solutions. Data is fitted using the Carreau-Yasuda model (solid lines).

The rheological properties of the polymeric test solutions are measured in steady shear at 22 °C using an Anton Paar MCR 502 stress-controlled rotational rheometer equipped with a 50 mm diameter 1° cone-and-plate geometry. The resulting flow curves of viscosity *η* as a function of the applied shear rate 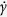 are shown in Fig. 4 in comparison with the viscosity of the pure solvent, *η*_*s*_ = 59 mPa s. The a-PS solutions are rather weakly shear-thinning over the shear rate range. The flow curves are fitted with a Carreau-Yasuda Generalized Newtonian Fluid (GNF) model^84^ from which the zero-shear viscosities of the fluids (*η*_0_) are extracted. The values obtained for *η*_0_ are provided in Table 2. Table 2 also includes the characteristic relaxation time of each fluid, *τ*, the determination of which is made from direct measurements of polymer stretching in the OSCER device itself and will be described in due course. For future computational studies, the molecular parameters and rheological information provided in Tables 1 and 2 also facilitate fitting of the data to a range of viscoelastic constitutive equations such as the White-Metzner model or the Finitely-Extensible Non-linear Elastic (FENE) dumbbell model, for example^84^. The value of Δ*n*_0_ in Table 2 refers to the magnitude of flow-induced birefringence that can be expected from a solution of polymer molecules fully stretched to their contour length. For a-PS in DOP it has been calculated that 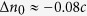, with *c* expressed in terms of the mass of polymer per unit mass of solution^25^,^69^.

### Viscoelastic flow in the OSCER

In the flow experiments performed using viscoelastic fluids in the OSCER device, the Weissenberg number is defined in the standard way, i.e. 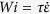, while the Reynolds number is calculated according to:


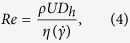


where 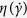 is the shear rate dependent viscosity found using the Carreau-Yasuda fit to the steady flow curves (Fig. 4) evaluated at a characteristic deformation rate 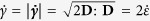. Since the polymer concentrations in the test solutions are quite low, the densities of the fluids do not vary significantly from that of the solvent, and we take *ρ* = 0.985 g mL^−1^ in all cases. In the experiments, the Weissenberg number is varied over a range 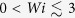, while the Reynolds number remains low (*Re* < 1). The elasticity number is given by *El* = *Wi*/*Re*. Since the polymeric test fluids are mildly shear-thinning (Fig. 4), the Reynolds number increases non-linearly, hence *El* decreases slightly with increasing shear rate.

**Figure 5:**
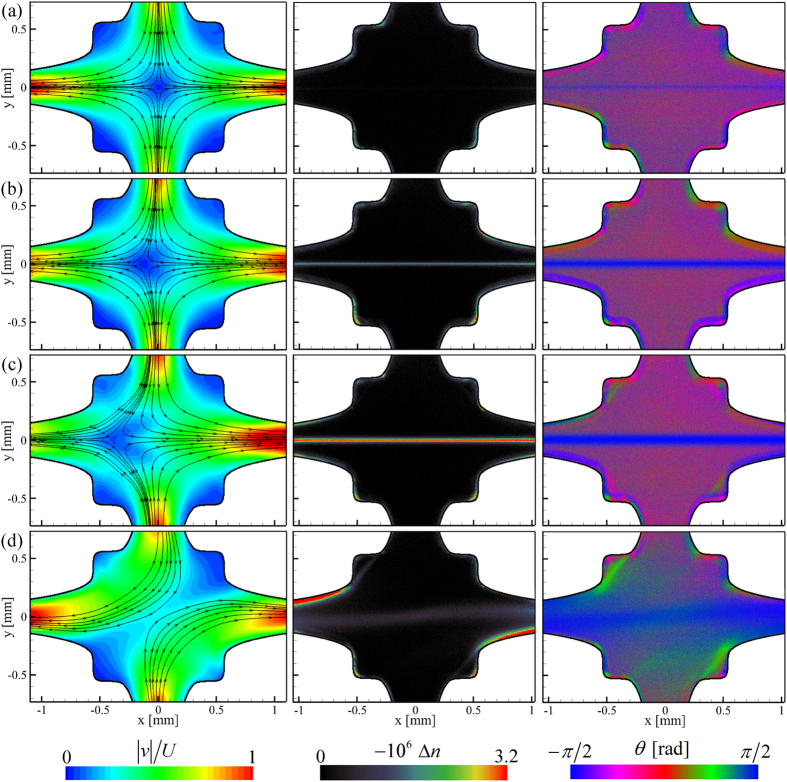
Example results from flow experiments conducted with one of the polymeric test fluids showing the evolution of velocity fields and flow-induced birefringence in the OSCER geometry for a 0.07 wt.% solution of PS16 in DOP as the flow rate (or *Wi*) is increased: (**a**) 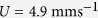, 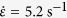, 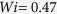, 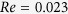. (**b**) 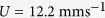, 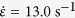, 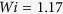, 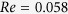. (**c**) 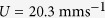, 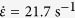, 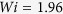, 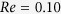. (**d**) 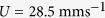, 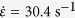, 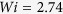, 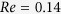. Left column: normalized velocity fields (time-averaged over two seconds) with superimposed streamlines; middle column: flow-induced birefringence; right column: angle of slow optical axis (*θ* = 0 radians corresponds to the *x*-direction). For polystyrene the slow axis is perpendicular to the direction of backbone chain orientation.

The viscoelastic a-PS in DOP solutions are examined in flow through the OSCER device over a range of imposed 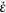 using a combination of flow-induced birefringence measurements and time-resolved *μ*-PIV. Figure 5 shows the evolution of flow patterns (here time-averaged over 2 s) and the spatial distribution of birefringence (Δ*n*) measured in the OSCER device for the flow of a 0.07 wt.% solution of PS16 as the imposed strain rate is increased. At lower 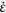 (Fig. 5a) the velocity field appears quite Newtonian-like, with a centrally-located stagnation point about which incoming streamlines divide symmetrically between the outlet channels. Here, the birefringence is at the lower end of the color scale, although there is in fact some degree of polymer chain alignment along the outflowing symmetry axis, as evidenced by the corresponding plot of the orientation angle of the slow optical axis, *θ*. In this plot, the blue coloration on the horizontal flow axis indicates orientation of the slow optical axis along the *y*-direction (*θ* = ±*π*/2 rad). This is consistent with the principal direction of polymer chain segment orientation, which indicates the axis for extraordinary polarizations, being along the outflow direction *χ* = 0 rad. This is because, due to the benzene-ring side groups, the refractive index of stretched polystyrene is greatest in the direction perpendicular to the direction of backbone orientation (i.e. for ordinary polarizations), resulting in a negative birefringence and a negative stress-optical coefficient^85^. As 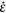 is increased (Fig. 5b) the velocity field begins to deviate from the Newtonian-like form; the stagnation point has been displaced laterally and the incoming streamlines bend left towards the displaced stagnation point. Birefringence is now clearly visible in the form of a localized strand aligned along the outflowing stagnation point streamline and the width of the band of oriented polymer has increased significantly. As 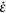 is increased further (Fig. 5c) the flow becomes more unstable and the stagnation point becomes more significantly displaced from the center point of the OSCER device. Interestingly, even under this severely distorted flow field, the birefringent strand appears to remain localized, uniform and unperturbed. Finally, at higher 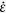 (Fig. 5d) a large scale symmetry-breaking results in a globally asymmetric flow field reminiscent of that previously reported in standard-shaped cross-slot devices^34^,^35^,^38^,^39^,^40^,^41^,^42^,^43^. Here the birefringence also exhibits asymmetry along with a significant reduction in apparent intensity.

The results displayed in Fig. 5 are quite representative of the evolution in flow behavior observed with all of the five different polymeric test solutions, except that the onset of different behavior occurs for fluid-dependent values of 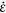. It is important to note that the lateral displacement of the stagnation point (as exemplified in Fig. 5b,c) can be either to the left or to the right of the centre point. We identify this distortion of the flow field with the onset of a first viscoelastic flow instability at *Wi* = *Wi*_*c*1_. Equally, the global asymmetry (exemplified in Fig. 5d) can be either clockwise or counterclockwise with respect to either one of the flow axes; we identify this as the onset of a second viscoelastic flow instability at *Wi* = *Wi*_*c*2_. It should also be remembered that the velocimetry fields shown in Fig. 5 are time-averaged over 2 seconds of flow. In fact once instability develops (i.e. Fig. 5b–d) the flow field exhibits increasing spatio-temporal fluctuations as 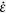 is increased. Discussion and analysis of these fluctuations will follow below, but it is important to note here that this fluctuation can have some effect on the birefringence measurements. The flow-induced birefringence images shown in Fig. 5 are formed from a combination of seven individual images, each captured with a 1 s exposure time under different modulation states of the light source (see Methods Section). Thus, for steady flows they can be considered as “time-averaged” over a total of approximately 7 s of flow. However, if the position of the birefringent strand fluctuates between the acquisition of the seven individual frames (as it certainly does in Fig. 5d) the final result should only be interpreted qualitatively.

We note that the lateral asymmetry and unsteady oscillatory motion of the stagnation point observed here for *Wi*_*c*1 _≤ *Wi *≤ *Wi*_*c*2_ is clearly distinct from the oscillatory instability reported by Varshney *et al*.^50^ for viscoelastic flow in a T-shaped junction with a recirculating cavity, and is also distinct from the inertio-elastic instabilities previously reported for the flow of weakly elastic fluids in the OSCER device^37^. In both of those previous cases the fluctuations were measured in the direction orthogonal to the direction of flow, whereas in the present case the periodic displacement of the stagnation point is along the outflow direction.

**Figure 6:**
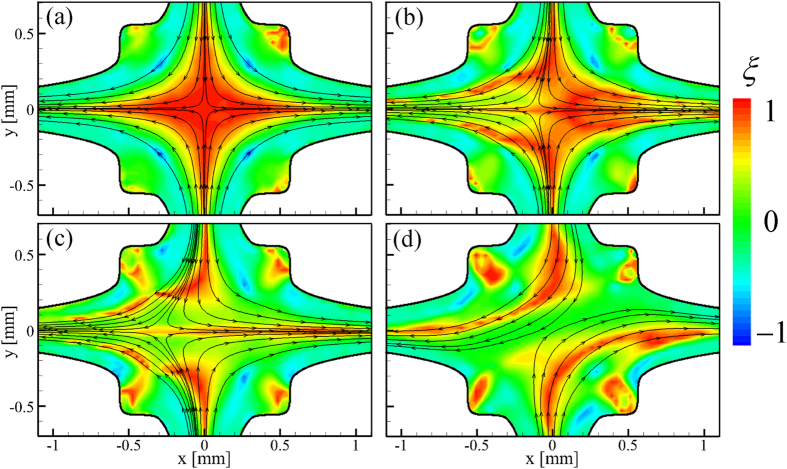
Spatially-resolved evaluation of the flowtype parameter ξ for flow of the 0.07 wt.% solution of PS16 in DOP at conditions equivelent to those in Fig. 5a–d, respectively. i.e. (**a**) 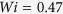, 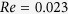. (**b**) 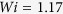, 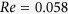. (**c**) 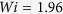, 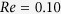. (**d**) 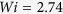, 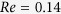. Analysis is performed on time-averaged velocity fields.

In Fig. 6a–d we use the time-averaged flow fields shown in Fig. 5a–d to evaluate the flowtype parameter (Equation 3) for the 0.07 wt.% solution of PS16 under the various flow regimes that were described previously. Under Newtonian-like flow conditions (Fig. 6a) the central region of the flow field is dominated by purely extensional kinematics and is quite comparable to the result for the Newtonian solvent at low *Re* (Fig. 3b). As the Weissenberg number is increased beyond *Wi*_*c*1_ and the stagnation point becomes increasingly displaced laterally (Fig. 6b,c) this central region becomes increasingly dominated by shear, although extensional flow persists along the horizontal flow axis passing through the stagnation point. For *Wi *> *Wi*_*c*2_ the global symmetry breaking causes complete loss of the stagnation point and the central strand of extensional flow is replaced by a region of purely shearing kinematics.

**Figure 7:**
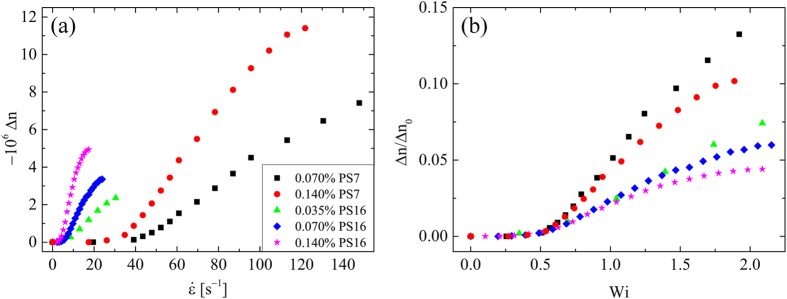
Measurement of flow-induced birefringence made at the location 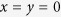 over a range of imposed flow rates enables the characteristic relaxation times *τ* of the polymer solutions to be determined: (**a**) birefringence Δ*n* as a function of 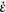 shows an increase for 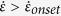. (**b**) Shifting the data to an onset of 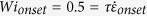 provides the relaxation time. Here, Δ*n* is normalized by the birefringence expected for a solution of fully-stretched molecules Δ*n*_0_, which can be used to estimate the degree of macromolecular deformation in each case. For each data series, the final data point shown at high 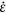 (or high *Wi*) represents the final measurement made before the onset of the global flow asymmetry, as exemplified in Fig. 5d.

For flow rates below this second transition (i.e. for *Wi *< *Wi*_*c*2_), we measure the value of Δ*n* at the location *x* = *y* = 0 as a function of the imposed strain rate, see Fig. 7a. The birefringence begins to increase as 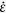 is increased beyond an onset value 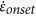. This onset can be shifted to 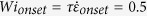 (Fig. 7b) in order to obtain the characteristic relaxation times of the polymer solutions, *τ*, provided in Table 2. In Fig. 7b we have normalized the measured birefringence by that expected from solutions of fully-stretched polymer molecules, Δ*n*_0_ = −0.08*c*, Table 2 25. This normalized birefringence can be related to the ensemble-averaged end-to-end length of polymer chains 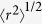 through the model provided by Treloar^25^,^86^, which relates the optical properties of strained polymeric networks to the mean segmental orientation. Based on this model, we estimate the polymer stretches to reach an ensemble-average end-to-end separation of 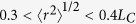 before the onset of the global flow asymmetry at *Wi*_*c*2_.

### Analysis of time-dependent viscoelastic flow in the OSCER

In the viscoelastic polystyrene solutions, as the imposed strain rate is increased, and flow instability develops, the flow becomes time-dependent and exhibits an increasing degree of spatio-temporal fluctuation.

**Figure 8:**
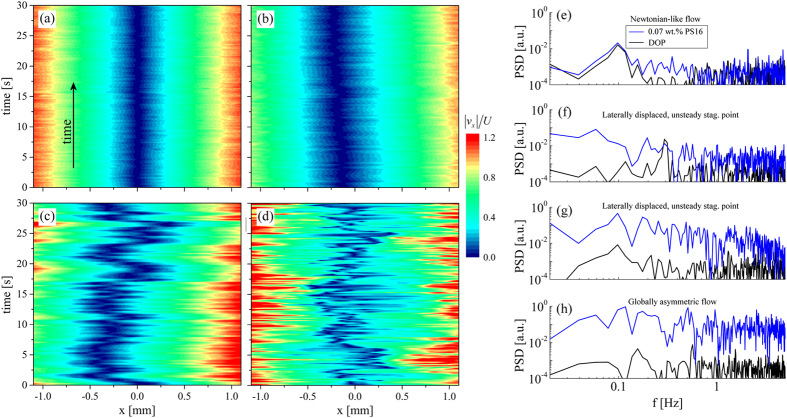
Time-resolved flow velocimetry illustrates the progressive increase in fluctuations as the *Wi* is increased. Space-time diagrams show |*v*_*x*_| along *y* = 0 (normalized by *U*) for flow of a 0.07 wt.% solution of PS16 in DOP under the following conditions: (**a**) 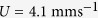, 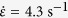, 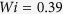, 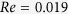. (**b**) 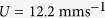, 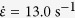, 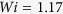, 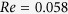. (**c**) 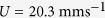, 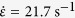, 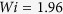, 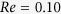. (**d**) 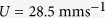, 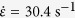, 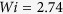, 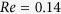. Movies M1,M2,M3–M4 in the Electronic Supplementary Information show full 2D, spatio-temporally-resolved velocity fields corresponding to Fig. 8a–d, respectively. (**e–h**) Power spectral density (PSD) of normalized velocity signals 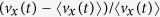 made at 10 Hz over a 30 s time period at location *x* = 1 mm, *y* = 0 mm under flow conditions corresponding to parts a–d, respectively. The PSD obtained for velocity signals from the DOP solvent at equivalent flow rates is also shown for comparison.

In Fig. 8 we represent this time-dependence in the form of space-time diagrams constructed from profiles of 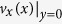 measured at a sampling rate of 4 Hz over a 30 s time period. The images show representative data obtained for the flow of the 0.07 wt.% solution of PS16 over a range of flow rates spanning the regimes of Newtonian-like flow (Fig. 8a), laterally-displaced unsteady stagnation point (Fig. 8b,c), and globally asymmetric unsteady flow (Fig. 8d). Movies showing full 2D, spatio-temporally-resolved velocity fields corresponding to Fig. 8a–d, are provided in the Electronic Supplementary Information as Movies M1,M2,M3–M4, respectively.

We analyse the power spectral density (PSD) of the velocity fluctuations for each test fluid over time at a location *x* = 1 mm, *y* = 0 mm, using a normalized velocity magnitude, 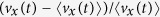, where <> represents a time-average. For this analysis, data was captured at a rate of 10 Hz for 30 s. Representative results from the 0.07 wt.% solution of PS16 under flow conditions corresponding to the cases shown in Fig. 8a–d are provided in Fig. 8e–h, respectively. For comparison, in Fig. 8e–h we also show PSD’s corresponding to velocity magnitude fluctuations occurring for the flow of the Newtonian solvent at equivalent flow rates. In the Newtonian-like flow regime, Fig. 8e, the PSD measured with the polymer solution is virtually indistinguishable from that of the Newtonian solvent and fluctuations are extremely low. For the solvent, the fluctuations remain small as the flow rate is increased. However for the polymer solution, as the Weissenberg number increases above *Wi*_*c*1_ and the stagnation point begins to exhibit lateral displacements and unsteadiness, some significant peaks in the PSD rise above the base level noise (Fig. 8f,g). For *Wi *> *Wi*_*c*2_ (Fig. 8h), broadband velocity fluctuations are clearly evident in the PSD. The sequence of behaviors demonstrated by Fig. 8 is typical of all the five polymer solutions we examined. Although velocity fluctuations become easily detectable as the flow becomes increasingly unstable, the power spectra are complex and no distinct characteristic frequencies are manifested.

### Identification of *Wi*_c1_

Further analysis of temporal velocity fluctuations in the flowing fluids is performed by evaluating the turbulence intensity along the *x*-axis, 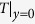. The turbulence intensity is defined by:


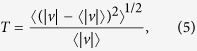


where 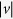 is the velocity magnitude at a particular 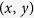 location and 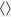 represents an average over all frames in a particular time series of velocity vector fields.

**Figure 9:**
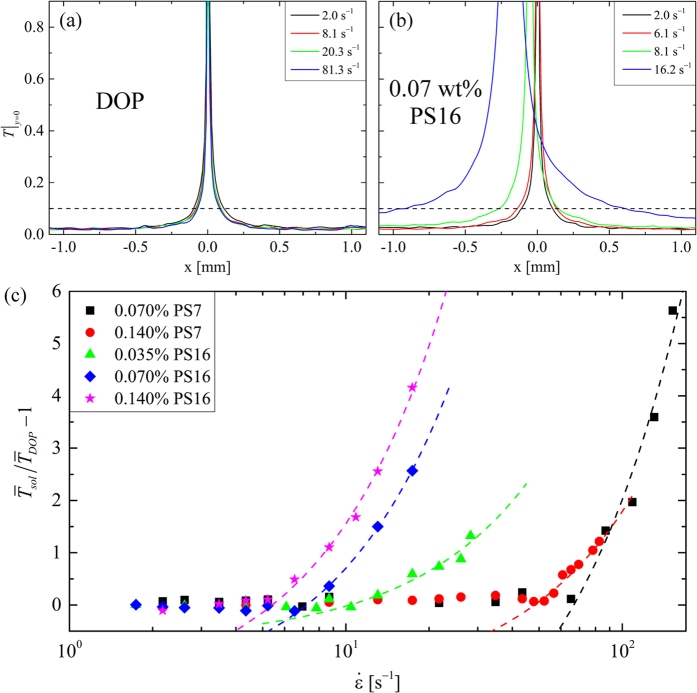
Determination of critical onset conditions for the first elastic flow instability using measurements of the turbulence intensity along the *x*-axis, 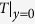. (**a**) For the Newtonian solvent, the turbulence intensity along *y* = 0 is independent of the applied strain rate. (**b**) For polymer solutions, the onset of instability results in a large increase in *T* due to lateral motion of the stagnation point. Average values, 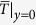, are obtained for both the solvent and the polymer solutions and are compared in (**c**). The large peak for 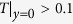, above the dashed lines in (**a**,**b**), is omitted from the average since variability in its size can skew the result. (**c**) The onset of instability at 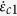 is determined by linear extrapolation of the growing region of the curve to 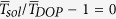. Dashed lines in (**c**) are linear fits to each data set of the form 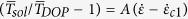.

Figure 9a,b shows how *T* varies with position along the *x*-axis for flow of the Newtonian solvent and the 0.07 wt.% PS16 solution, respectively, over a range of imposed 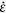. For the DOP solvent Fig. 9a, *T* is basically independent of the flow rate. Note the large peak in *T* for the Newtonian fluid at *x* = *y* = 0 (Fig. 9a) arises because here at the stagnation point velocity fluctuations, though small, remain finite but the time-average value of 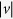 is close to zero. In the polymer solution (Fig. 9b) as 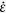 increases such that *Wi *> *Wi*_*crit*1_, lateral displacement of the stagnation point means this peak in *T* may move off-center and increasing fluctuations in 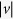 mean that the peak broadens significantly. (Note that for the polymer solutions, the magnitude of the peak in *T* generally increases with increasing 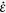, however this measure has poor reproducibility due to the sharpness of the peak and to the spacing between velocity vectors obtained from the *μ*-PIV). Avoiding values of *T *> 0.1 (above the horizontal dashed lines in Fig. 9a,b), we obtain average values of 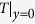 for both the polymer solutions (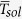) and the pure DOP (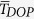) which we compare as a function of 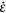 in Fig. 9c. At low imposed strain rates, velocity fluctuations in the polymer solutions are similar to the solvent so that 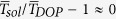. Above a fluid-dependent critical strain rate 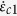, velocity fluctuations in the polymer solutions begin to grow relative to fluctuations in the DOP and non-zero values of 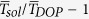 are obtained. For 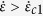 linear growth of (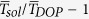) with strain rate is observed. The dashed curves in Fig. 9c are linear fits to the data of the form 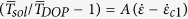, from which the value of 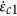 for each fluid is obtained. This allows an unambiguous value of the first critical Weissenberg number to be obtained by 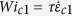. Of course, the second critical Weissenberg number (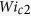) is quite easy to identify since the transition to the globally asymmetric flow state is very obvious. Values of *Wi*_*c*1_ and *Wi*_*c*2_ are provided in Table 3 along with corresponding values of *Re*_*c*1_ and *Re*_*c*2_ determined from Eq. 4.

### Summary of results in dimensionless parameter space

**Figure 10:**
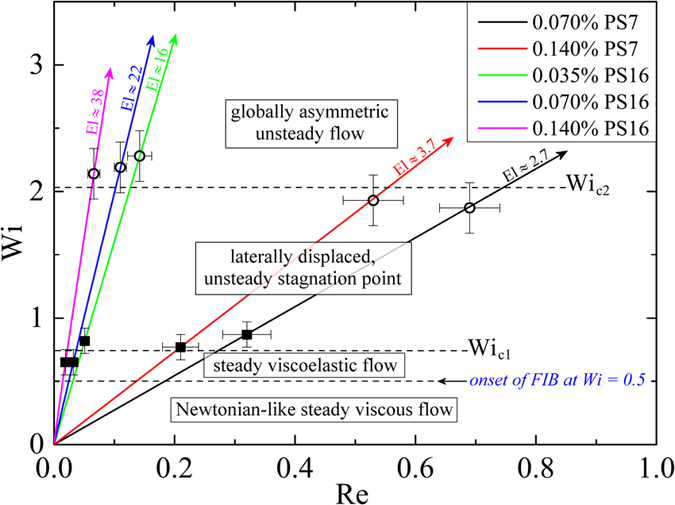
Stability diagram in dimensionless *Wi-Re* state space for the onset of viscoelastic instabilities during ideal planar elongational flow in the OSCER device. Closed squares represent *Wi*_*c*1_ and open circles represent *Wi*_*c*2_. Error bars on data points represent the typical ranges of *Wi* and *Re* for the onset of instabilities in each polymer solution over at least five experimental test runs in each case.

In Fig. 10, we summarize the onset of different flow regimes in the OSCER device using a dimensionless *Wi*–*Re* parameter space. Here, the colored lines with arrows represent the trajectories of different polymer solutions with different elasticity numbers (*El* = *Wi*/*Re*) through this dimensionless state space. Since the fluids are only mildly shear-thinning, the approximate values of *El* shown in Fig. 10 and listed in Table 3 are simply obtained using Reynolds numbers based on the measured zero shear viscosities of the fluids. For flow at *Wi *< *Wi*_*onset*_ = 0.5, Fig. 10 shows the regime of Newtonian-like steady viscous flow. For 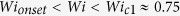, the polymer begins to stretch significantly in the flowfield, but the flow remains steady and here we define a regime of “steady viscoelastic flow”. As the Weissenberg number is increased such that *Wi *> *Wi*_c1_ ≈ 0.75, the flow transitions to the state of the laterally displaced, unsteady stagnation point. Finally, for *Wi *> *Wi*_*c*2_ ≈ 2 the flow transitions to the globally asymmetric unsteady flow state.

### Evaluation of the elastic instability criterion *M*

**Figure 11:**
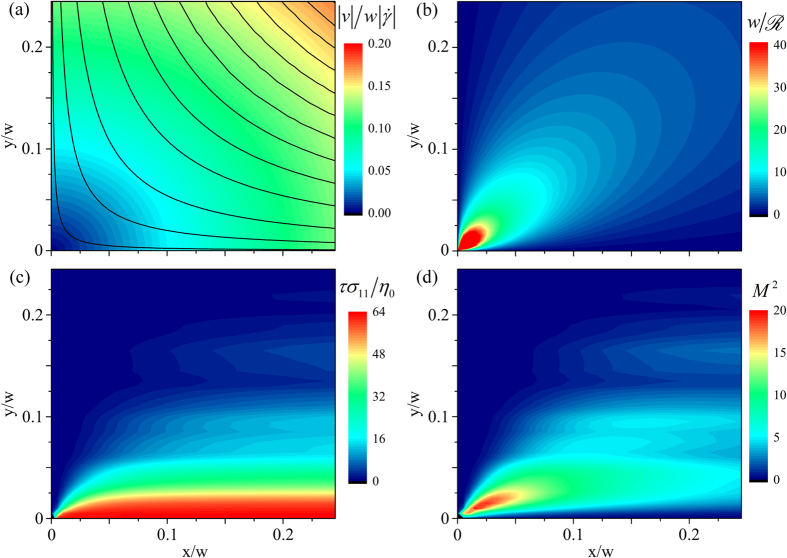
Determination of the elastic instability criterion *M*^2^ at the onset of the first elastic instability at (*Wi*_*c*1,_*R*e_*c*1_) for the 0.035 wt.% solution of PS16 in the OSCER device. (**a**) dimensionless velocity field and hyperbolic streamlines determined using the ideal stream function for planar elongational flow. (**b**) dimensionless streamline curvature. (**c**) dimensionless streamwise stress determined from birefringence measurements made at *Wi* = 0.7, *Re* = 0.04. (**d**) Spatial distribution of *M*^2^ values obtained by combining data in parts (**a**) to (**c**) according to Eq. 1.

We evaluate the elastic instability criterion *M* or *M*^2^ (Equation 1) at conditions as close as possible to the onset of the first instability at *Wi*_*c*1_. Since a combination of both streamline curvature and streamwise stress is required to obtain non-zero values of *M*, we perform the analysis over a small quadrant near the stagnation point, corresponding to 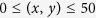 *μ*m. For 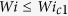, since the flow field only deviates weakly from the Newtonian case (see e.g. Figs 2b, 3b, 5a and 6a), we assume the flow field is well represented by the ideal streamfunction 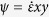. This assumption should also be most valid away from the confining walls of the OSCER device and close to the stagnation point, where we perform this evaluation (see Fig. 3). Velocity components are given by 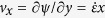, 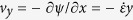 and the local velocity magnitude is 
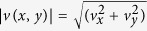
. The velocity magnitude is shown in dimensionless form in Fig. 11a with superimposed streamlines determined from the streamfunction. The curvature of streamlines at any point in space is given by the following general expression:


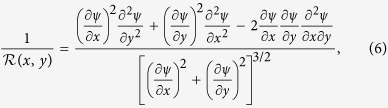


which, for ideal planar extensional flow, can be simplified to:


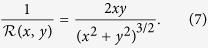


The streamline curvature is shown in dimensionless form over the 50 × 50 μm quadrant in Fig. 11b.

The elastic tensile stress along streamlines *σ*_11_ is estimated using the following procedure. Firstly, the components of the stress tensor **σ** are estimated by applying the stress-optical rule (SOR)^24^ to the spatially-resolved measured birefringence, Δ*n*, see Fig. 5. The stress-optical rule states that Δ*n* and the components of **σ** obey the following relations:


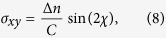


and


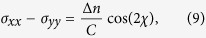


where *C* is the stress-optical coefficient^24^. For moderate polymer deformations and stresses, the value of *C* is often found to be constant for a given polymeric system. In our experiments, at 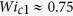 we estimate the ensemble-average end-to-end separation of polymer chains in the elastic strand to be 
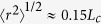
25,86. Previous experiments with similar fluids in stagnation point flows have shown a linear dependence between stress and birefringence up to much higher polymer extension than this^26^,^87^, so here we assume linearity of the SOR. In the case of polystyrene, several previous studies have measured values of *C* in a close range between 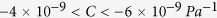
26,88,89,90, here we take a representative value of 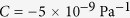
91. We make a further approximation by assuming that 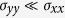. This seems reasonable given that the deformation occurs predominantly along the *xx* direction, as clearly shown by Figs 3 and 5, as well as in previous works^27^.

Since the polymer orientation angle in the birefringent strand is along 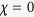 rad (Fig. 5), we find 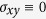 and the stress tensor only contains a single non-zero component given by 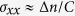. The streamwise tensile stress can then be found as follows:


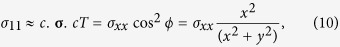


where **c** is the direction-cosine transformation matrix and 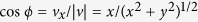. A spatial map of dimensionless *σ*_11_ values (scaled by an elastic modulus 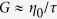) thus determined from birefringence measurements made with the 0.035 wt.% solution of PS16 at 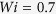 is provided in Fig. 11c. Finally the data shown in Fig. 11a–c can be combined on a pixelwise basis according to Eq. 1 in order to obtain a spatially-resolved map of the instability criterion *M*^2^ in the region of the stagnation point at conditions close to the onset of instability at *Wi*_*c*1_, as shown in Fig. 11d. We observe contours of *M*^2^ of similar form to those shown numerically by Öztekin *et al*.^72^ for planar stagnation point flow of viscoelastic fluids modelled by the Oldroyd-B constitutive equation. In our experiment with the 0.035 wt.% PS16 solution, we find a maximum value for the instability criterion 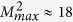 is reached at a location close to the stagnation point. More precisely, we report a mean and standard deviation value of 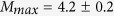 over a 3 × 3 pixel area centered on the location 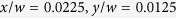.

**Figure 12:**
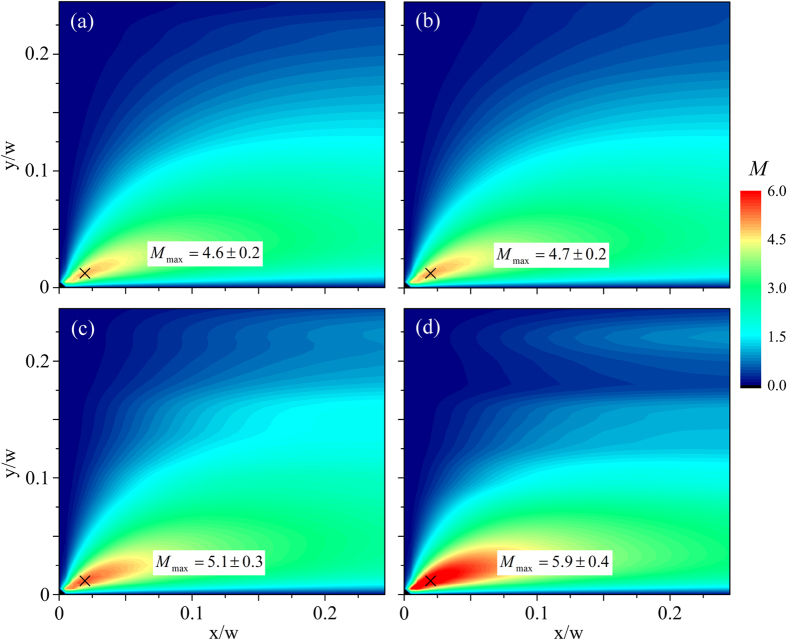
Spatially-resolved evaluation of *M* at conditions as close as possible to the onset of the first instability at (*Wi*_*c*1,_ *R*e_*c*1_) provides reasonable agreement between values for the various test fluids. (**a**) 0.07 wt.% PS7 at *Wi* = 0.90, *Re* = 0.33; (**b**) 0.14 wt.% PS7 at *Wi* = 0.74, *Re* = 0.20; (**c**) 0.07 wt.% PS16 at *Wi* = 0.69, *Re* = 0.03; (**d**) 0.14 wt.% PS16 at *Wi* = 0.63, *Re* = 0.02. In each case the maximum value, *M_max_*, is located at position 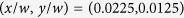, marked “×”, and the value anotated in each plot is an average over values of *M* obtained over a 3 × 3 pixel area (≈ 9 *μ*m^2^) centered on that point.

Rather similar results are obtained from the remaining four polystyrene-based test solutions, see Fig. 12. We equate the resulting values of *M*_*max*_ in the spatial maps of the instability criterion with the value of *M*_*crit*_ for the onset of elastic instability. For our test solutions, *M*_*crit*_ varies in a narrow range 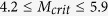, indicating that the geometric and rheological scaling of *M* provided by Eq. 1 holds well for this planar elongational flow. The values of *M* and their standard deviations obtained for individual fluids are shown in Table 3. These values of *M* are of similar magnitude to those predicted numerically by Öztekin *et al*.^72^ in planar elongational flow as well as to previously reported values determined from experiments performed in viscometric torsional shearing flows^5^.

## Discussion and Conclusions

In this work we have employed a series of very well characterized viscoelastic polymer solutions to examine the onset of elastically-induced flow instabilities in an almost ideal planar stagnation flow in an optimized-shape cross-slot extensional rheometer (OSCER). We have measured the growth of flow-induced birefringence and performed time-resolved flow velocimetry measurements on the fluids as the dimensionless Weissenberg number is increased by control of the total volume flow rate through the microfluidic OSCER device. As *Wi* increases above *Wi*_*onset*_ = 0.5 the flow field remains steady and Newtonian-like as a narrow, localized birefringent strand develops along the outflowing symmetry axis of the flow. As the Weissenberg number increases above *Wi*_*c*1_ ≈ 0.75, we observe a new type of elastic instability characterized by a lateral displacement and local unsteadiness of the stagnation point. In this instability, the stagnation point moves erratically, and apparently quasiperiodically, from side to side along the ouflowing symmetry axis of the flow device. This is in contrast to other recently-reported oscillatory viscoelastic flow instabilities, in which the periodic fluctuations were observed in the direction perpendicular to the outflow axis^37^,^50^. At a higher critical Weissenberg number *Wi*_*c*2_ ≈ 2, a second instability results in the flow breaking symmetry globally in a manner that resembles previously reported elastic flow asymmetries in standard-shaped cross-slot devices^34^,^35^,^38^,^39^,^40^,^41^,^42^,^43^. A similar sequence corresponding to a local time-dependent flow instability preceeding a global elastic instability has also been documented along strongly curved streamlines near the re-entrant corner of an abrupt contraction geometry^53^.

At conditions close to *Wi*_*c*1_, we have used the birefringence measurements from our extensional flow experiments to evaluate a well-known criterion for the onset of elastic instabilites^5^,^66^,^72^. We have found maximum values of the criterion, *M*_*max*_, occur close to the stagnation point where there is a critical combination of high tensile viscoelastic stress, strongly curved streamlines and non-zero flow velocity. Values of *M* at this location are essentially fluid independent and have a magnitude similar to critical values of *M* reported at the onset of elastic instabilities in well-defined torsional shearing flows. Our experimental results thus support the arguments of Öztekin *et al*.^72^ that the mechanism for the onset of elastic instability in planar stagnation point flow is similar to that for elastic instabilities in simple shearing flows. That is to say, coupling between streamline curvature and streamwise elastic tensile stresses results in the amplification of small disturbances to the base flow. This mechanism appears to be independent of whether the curvature and stresses arise due to shearing or extensional kinematics and should thus be applicable to complex mixed flows with arbitrary kinematics. These observations, taken in conjunction with recent arguments by James^92^ regarding the magnitude of elastic normal stresses in a wide range of complex flows with mixed kinematics, and measurements of oscillatory instabilities in other flow geometries^37^,^50^ suggest that the onset of spatially-localized viscoelastic instabilities may indeed be ubiquitous at moderate values of the Weissenberg number.

In this work we have focused on relatively simple viscoelastic fluids with almost constant viscosity. In the future it will be interesting to examine the influence of shear thinning on the critical onset conditions for the observed flow transitions. Sousa *et al*.^42^ have recently studied the onset conditions for the forward bifurcation leading to the global flow asymmetry in standard-shaped microfluidic cross-slots using fluids with a wide range of rheological properties. They found a general trend for a reduction in the critical Weissenberg number as the degree of fluid shear thinning increased. Strongly shear thinning fluids tended to transition to a steady asymmetric flow state, while less shear thinning fluids displayed a tendency to transition to a time-dependent asymmetric state^42^ (which is consistent with the observations reported here for flows above *Wi*_*c*2_). By contrast, recent experiments and numerical simulations of viscoelastic flows in serpentine microchannels indicate that in this shear-dominated geometry, shear thinning may have a stabilizing effect on the onset of purely elastic instabilities^93^. Unraveling the connections between the local flow kinematics, the fluid rheology and the mode of instability should become possible in the near future by combining these detailed microfluidic observations (all performed using well-characterized polymer solutions) with recent developments in computational abilities for studying time-dependent and three-dimensional viscoelastic flows^42^,^43^,^75^,^93^,^94^.

## Materials and Methods

### Polymer solution preparation

Polymer solutions are prepared using an intermediate solvent method. The polystyrene powder is weighed and dissolved in a small quantity (≈ 20 mL) of dichloromethane. After complete dissolution, the polystyrene plus dichloromethane mixture is added to an appropriate volume of the final solvent dioctyl phthalate (DOP). The fluids are mixed by gentle hand swirling until no refractive index variations can be seen throughout the mixture. Finally the dichloromethane is removed by evaporation in a fume hood maintained at room temperature. The removal of the dichloromethane is monitored by periodic weighing and is considered complete when there is no further weight loss.

### Microdevice fabrication

The microfluidic OSCER device is fabricated by cutting channels through 2 mm thick stainless steel by the technique of wire-electrical discharge machining with a 30 *μ*m diameter copper wire. Subsequently, annealed soda-glass windows are bonded (using silicone aquarium adhesive) to the upper and lower flat surfaces of the stainless steel in order to form enclosed channels with optical access to the inside. One of the glass windows has four holes drilled through it ultrasonically, through which fluid can be injected or withdrawn appropriately in order to drive the flow through the device. This technique allows the fabrication of high-aspect ratio microfluidic devices in materials amenable to use with organic solvents, able to resist deformation under high pressures, and with high quality optical access so as to reduce background noise in sensitive flow-induced birefringence measurements. Additional details of this fabrication technique are provided in several previous works^26^,^49^.

### Flow-induced birefringence measurement

Flow-induced birefringence measurements are performed using an Exicor MicroImager^*TM*^ (Hinds Instruments, Inc., OR). For these measurements, a light emitting diode sends collimated monochromatic light (wavelength *λ* = 535 nm) along an optical line consisting of (a) a linear polarizer at 0°, (b) a photoelastic modulator (PEM) at 45°, (c) a PEM at 0° and (d) a linear polarizer at 45°. The sample (in this case the OSCER device containing polymer solution) is positioned on the imaging stage of the instrument between the two PEMs. A 5× objective lens is used to focus light from the midplane of the OSCER device onto a 2048 × 2048 pixel, 12-bit CCD array (which provides a field of view ≈ 2 × 2 mm and hence a spatial resolution of ≈ 1 *μ*m/pixel). A stroboscopic illumination technique^95^,^96^ is used to determine the elements of the 4 × 4 Mueller matrix *P* necessary to compute the pixelwise sample retardance *δ* and angle of the high refractive index (i.e. slow) optical axis *θ* over the full field of view:


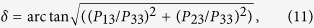


and


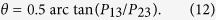


To compute the required elements of the Mueller matrix, the CCD camera records a total of seven frames, each accumulated over ≈1 s, and each with the light source modulated in order to sample specific polarization states achieved within the PEM cycle^96^. The spatially-resolved birefringence of the sample is obtained as follows:


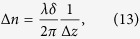


where Δ*z* is the optical pathlength through the sample. In the case of fluid flow in the OSCER geometry, due to the high aspect ratio (*α* = 10) we assume two-dimensional flow and equate Δ*z* with the depth of the geometry, *d*.

### Flow velocimetry

Micro-particle image velocimetry (*μ*-PIV) is performed by seeding the test fluids with 2 *μ*m diameter fluorescent melamine resin tracer particles (MF-FluoOrange-1240, microParticles GmbH, Germany) with excitation/emission wavelengths of 560/584 nm. The particle concentration in the test fluid is *c*_*p*_ ≈ 0.02 wt.%. The imaging system consists of a 1280 × 800 pixel, CMOS camera (Phantom Miro M310, Vision Research Inc., NJ), capable of acquiring image pairs for PIV analysis at up to 1600 Hz, and an inverted microscope (Nikon Eclipse TE 2000). A 4×, NA  =  0.13 numerical aperture objective is used to focus on the midplane of the flow geometry. The resulting measurement depth over which microparticles contribute to the determination of the velocity field is 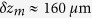
97, or ≈ 0.08*d*. The fluid is illuminated by a dual-pulsed *λ* = 527 nm Nd:YLF laser (Terra PIV, Continuum Inc., CA) with pulse width 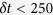 ns. The fluorescent seed particles are excited by the laser light and emit at a longer wavelength. The reflected laser light is filtered out with a G-2A epifluorescent filter, so that only the light emitted by fluorescing particles is imaged on the light sensor array. Images are captured in pairs with a time separation Δ*t* that is adjusted for each applied flow rate in order to always achieve an average particle displacement of approximately four pixels, optimal for subsequent PIV analysis. At each imposed flow rate, image pairs are captured at rates of both 4 and 10 Hz over a period of 30 s. The standard cross-correlation PIV algorithm (TSI Insight 4G software), with interrogation areas of 32 × 32 pixels and Nyquist criterion, is used to analyze each individual image pair to obtain sets of time-resolved flow fields. Tecplot Focus software (Tecplot Inc., WA) is used for further analysis of the velocity vector fields, i.e. to extract velocity profiles, perform averaging of flow fields, and to generate contour plots and streamline traces.

## Additional Information

**How to cite this article**: Haward, S. J. *et al*. Elastic instabilities in planar elongational flow of monodisperse polymer solutions. *Sci. Rep.* **6**, 33029; doi: 10.1038/srep33029 (2016).

## Supplementary Material

Supplementary Information

Supplementary Movie S1

Supplementary Movie S2

Supplementary Movie S3

Supplementary Movie S4

## Figures and Tables

**Table 1:** Molecular parameters of the a-PS samples under *θ*-solvent conditions.

a-PS sample	*M*_*p*_[MDa]	*M*_*w/*_*M*_*n*_	n	*L*_*C*_ [*μ*m]	*N*	*l_p_*[nm]	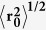 [nm]	*R*_g_[nm]	*L*^2^	c* [wt.%]
PS7	6.9	1.09	66346	16.6	6840	2.43	201	82	6821	0.5
PS16	16.2	1.07	155769	38.9	16059	2.43	308	126	15951	0.32

The peak molecular weight is *M*_*p*_ and the sample polydispersity is given by *M*_*w*_/*M*_*n*_, where *M*_*w*_ and *M*_*n*_ are the weight and number averaged molecular weights, respectively. The number of repeat units is *n* = *M*_*p*_/*m*, where *m* = 104 Da is the monomer molecular weight. The contour length *L*_*C*_ = *nl*_*m*_, where *l*_*m*_ = 0.25 nm is the monomer length. The characteristic ratio 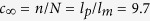, where *N* is the number of equivalent segments in an ideal chain and *l*_*p*_ is the persistence length. The ensemble-averaged equilibrium end-to-end distance of the random coil is 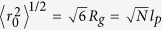, where *R*_*g*_ is the equilibrium radius of gyration. An extensibility parameter can be defined as 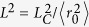. The characteristic concentration for overlap of polymer chains is found using the formula 
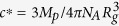
, where *N*_*A*_ is Avogadro’s constant^83^.

**Table 2:** Properties of the viscoelastic a-PS in DOP test solutions at various polymer concentrations.

property	PS7	PS7	PS16	PS16	PS16
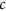 [wt.%]	0.07	0.14	0.035	0.07	0.14
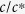	0.14	0.28	0.11	0.22	0.44
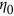 [mPa s]	71	81	68	82	107
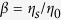	0.84	0.75	0.88	0.72	0.56
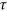 [ms]	13	16	80	90	120
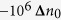	56	112	28	56	112

**Table 3:** Critical onset conditions for elastic instabilities in the OSCER device.

property	PS7	PS7	PS16	PS16	PS16
*c* [wt.%]	0.07	0.14	0.035	0.07	0.14
*El*	2.7	3.7	16.0	22.1	37.7
*Wi*_*c*1_	0.87	0.77	0.82	0.65	0.65
*Re*_*c*1_	0.32	0.21	0.05	0.03	0.02
*Wi*_*c*2_	1.85	1.93	2.28	2.19	2.14
*Re*_*c*2_	0.69	0.53	0.14	0.11	0.07
*M*_*crit*_	4.6 ± 0.2	4.7 ± 0.2	4.2 ± 0.2	5.1 ± 0.3	5.9 ± 0.4
