# Supplementary material for: Elastic instabilities in planar elongational flow of monodisperse polymer solutions
Source: Sci Rep. 2016 Sep 12;6:33029. doi: 10.1038/srep33029 (PMC5018825; doi:10.1038/srep33029)
Supplement: Supplementary Information [file srep33029-s1.pdf]

Elastic instabilities in planar elongational flow of monodisperse polymer solutions

Simon J. Haward[1,*], Gareth H. McKinley[2], and Amy Q. Shen[1]

[1]Okinawa Institute of Science and Technology, Onna-son, Okinawa, 904-0495, Japan
[2]Massachusetts Institute of Technology, 77 Massachusetts Avenue, Cambridge, MA 02139, United States
*simon.haward@oist.jp

Supplementary Information

List of Video Captions

Movie 1 (M1.mov): Time-resolved flow velocimetry for flow of a 0.07 wt% solution of PS16 in DOP at Wi = 0.39, Re = 0.019, and corresponding to the space-time data shown in Fig. 8(a) of the main article.

Movie 2 (M2.mov): Time-resolved flow velocimetry for flow of a 0.07 wt% solution of PS16 in DOP at Wi = 1.17, Re = 0.058, and corresponding to the space-time data shown in Fig. 8(b) of the main article.

Movie 3 (M3.mov): Time-resolved flow velocimetry for flow of a 0.07 wt% solution of PS16 in DOP at Wi = 1.96, Re = 0.10, and corresponding to the space-time data shown in Fig. 8(c) of the main article.

Movie 4 (M4.mov): Time-resolved flow velocimetry for flow of a 0.07 wt% solution of PS16 in DOP at Wi = 2.74, Re = 0.14, and corresponding to the space-time data shown in Fig. 8(d) of the main article.
